# Perinatal Women’s Perspectives of, and Engagement in, Digital Emotional Well-Being Training: Mixed Methods Study

**DOI:** 10.2196/46852

**Published:** 2023-10-17

**Authors:** Jacqueline A Davis, Jeneva L Ohan, Sonia Gregory, Keerthi Kottampally, Desiree Silva, Susan L Prescott, Amy L Finlay-Jones

**Affiliations:** 1 Telethon Kids Institute Nedlands Australia; 2 Medical School (Paediatrics) University of Western Australia Crawley Australia; 3 School of Medical and Health Sciences Edith Cowan University Joondalup Australia; 4 Nova Institute for Health Baltimore, MD United States

**Keywords:** perinatal, digital mental health interventions, well-being, mindfulness, self-compassion, engagement, ORIGINS

## Abstract

**Background:**

Psychological distress in the early postpartum period can have long-lasting deleterious effects on a mother’s well-being and negatively affect her infant’s development. Intervention approaches based in contemplative practices such as mindfulness and loving-kindness and compassion are intended to alleviate distress and cultivate well-being and can be delivered effectively as digital mental health interventions (DMHIs).

**Objective:**

To understand the feasibility of engaging perinatal women in digital interventions, this study aimed to document participants’ experiences in the Mums Minds Matter (MMM) study, a pilot randomized controlled trial comparing mindfulness, loving-kindness and compassion, and progressive muscle relaxation training delivered in a digital format and undertaken during pregnancy. To assess the different stages of engagement during and after the intervention, we adapted the connect, attend, participate, enact (CAPE) framework that is based on the idea that individuals go through different stages of engagement before they are able to enact change.

**Methods:**

The MMM study was nested within a longitudinal birth cohort, The ORIGINS Project. We aimed to recruit 25 participants per randomization arm. Data were collected sequentially during the intervention through regular web-based surveys over 8 weeks, with opportunities to provide regular feedback. In the postintervention phase, qualitative data were collected through purposive sampling.

**Results:**

Of 310 eligible women, 84 (27.1% [*connect* rate]) enrolled to participate in MMM. Of the remaining 226 women who did not proceed to randomization, 223 (98.7%) failed to complete the baseline surveys and timed out of eligibility (after 30 weeks’ gestation), and 3 (1.3%) displayed high psychological distress scores. Across all program groups, 17 (20% [*attend* rate]) of the 84 participants *actively* opted out, although more may have disengaged from the intervention but did not withdraw. The main reasons for withdrawal were *busy life* and *other priorities*. In this study, we assessed active engagement and ongoing skills use (*participate* and *enact*) through postintervention interviews. We undertook 15 participant interviews, conducted 1 month to 3 months after the intervention. Our results provide insights into participant barriers and enablers as well as app changes, such as the ability to choose topics, daily reminders, case studies, and diversity in sounds. Implementing a DMHI that is brief, includes frequent prompts or *nudges*, and is easily accessible is a key strategy to target perinatal women.

**Conclusions:**

Our research will enable future app designs that are sufficiently nuanced to maximize the uptake, engagement, and application of mental health skills and contemplative practices in the perinatal period. Providing convenient access to engaging and effective prevention programs is critical and should be part of prenatal self-care. Our research underscores the appeal and feasibility of digital intervention approaches based in contemplative practices for perinatal women.

**Trial Registration:**

Australian New Zealand Clinical Trials Registry (ANZCTR) 12620000672954p; https://anzctr.org.au/Trial/Registration/TrialReview.aspx?ACTRN=12620000672954p

**International Registered Report Identifier (IRRID):**

RR2-10.2196/19803

## Introduction

### Mental Health Promotion in the Perinatal Period

Anxiety and depression are the most common postpartum difficulties experienced by women globally [1], with an average global prevalence rate of >17% [2]. Maternal psychological distress (stress, anxiety, and depression) in the early postpartum period can disrupt a mother’s well-being and functional ability as well as interpersonal relationships, including those with her infant [3]. This reinforces a critical need for universal mental health promotion efforts that complement targeted treatment programs and span prevention through to early intervention [4]. To promote well-being and prevent perinatal mental health problems, the main focus of these initiatives should be in the prenatal period. Despite this, much existing practice concentrates on identification and treatment of mental health problems in the postnatal period, particularly postnatal depression [5].

A recent systematic review [6] indicated that mindfulness-based and multicomponent positive psychological interventions demonstrated the greatest efficacy in both clinical and nonclinical populations. Mindfulness-based interventions (MBIs) and compassion-based interventions (CBIs) are examples of contemplative interventions that target both alleviation of distress and cultivation of well-being [7-9]. Furthermore, research demonstrates that mindfulness and compassion are associated with meaningful outcomes for women and families during the perinatal period, such as better infant social-emotional development [10]. Accordingly, it has been suggested that contemplative interventions could be a useful component of mental health promotion strategies designed to support better outcomes for women and families.

### Digital Mental Health Interventions Based in Contemplative Practices

Given the need to provide scalable and low-cost support for women in the perinatal period, an important consideration for contemplative interventions is whether they can be taught effectively as digital interventions [11,12]. There are several potential benefits to delivering MBIs and CBIs in a digital format [13,14], such as the capacity to implement them cost-effectively and reach individuals who might struggle to access face-to-face services, as well as the ability to provide greater flexibility and universal access for service users [15]. However, it is currently unclear whether MBIs and CBIs delivered in a digital format are a feasible and efficacious means of reducing stress and promoting well-being in the perinatal period [16]. Available evidence for internet-delivered MBIs highlights some issues with intervention feasibility and engagement: a brief web-based self-compassion study for mothers of infants demonstrated efficacy but recommended further exploration in a randomized controlled trial (RCT) experimenting with delivery modalities that might enhance intervention uptake and engagement [17].

To better understand the feasibility of digital contemplative interventions for women in the perinatal period, we first need to understand how women progress through the program and when and why they discontinue. The Mums Minds Matter (MMM) study is a pilot RCT comparing 3 types of digital mental health interventions (DMHIs): mindfulness training (MT), loving-kindness and compassion training (LKCT), and progressive muscle relaxation training (PMRT; active control) for improving positive mental health and psychological distress among pregnant women [4]. Although interventions need to be efficacious, it is critical that participants engage in enough of the intervention to benefit. Furthermore, it is key that contemporary psychological treatments develop active skills in participants that can be practiced and sustained in applied ways, such as through *home practice* [18].

### DMHI Engagement and Application

Despite the increasing number of studies on digital interventions, few explore the construct of engagement, and there is a paucity of evidence of long-term skill use beyond the intervention period [19]. One framework for evaluating engagement in both face-to-face and digital programs is the connect, attend, participate, enact (CAPE) model [20]. This model identifies and defines 4 aspects of intervention engagement. First, *connect* is the number of people who express interest in engaging in an intervention out of those who are eligible. Second, *attend* relates to presence in an intervention, such as the initial and ongoing sessions that a participant completes (ie, perseverance). Third, *participate* is active engagement with intervention content, such as completing regular tasks and program continuity. Finally, *enact* refers to the participant applying intervention strategies or knowledge in daily life. Although the CAPE model was developed to guide face-to-face parenting program research, it can be adapted to understand and measure digital intervention engagement in a research context. The research team developed a logic model to aid the process of understanding how and when the CAPE framework can be applied to interpret the role of engagement variables in promoting outcomes in digital perinatal mental health and well-being programs [19]. The logic model is available in Multimedia Appendix 1.

This study is part of the feasibility assessment of MMM; the overall aim of this study is to characterize participant engagement and experience in a prenatal digital emotional well-being program to inform the design of a full-scale RCT. To assess the different stages of participant engagement, we adapted the CAPE model [20], which is used to assess engagement and is based on the idea that individuals must go through different stages of engagement before they are able to enact change.

The objectives of this study were as follows:

1. Assess and describe participant engagement and experience during and after the intervention.
2. Explore any engagement differences in the type of DMHI training provided (mindfulness vs loving-kindness and compassion vs PMRT).
3. Understand reasons for opting out of the intervention, including barriers and enablers.

## Methods

### Ethical Considerations

The study was conducted according to the guidelines of the Declaration of Helsinki. The MMM study was approved by the Ramsay Health Care WA|SA Human Research Ethics Committee (1927). It received ethics reciprocal approval from the University of Western Australia (2020/ET000334). Informed consent was obtained from all participants involved in the study.

### Setting

This study was nested within a longitudinal prospective pregnancy cohort study (The ORIGINS Project, hereinafter referred to as *ORIGINS*) tracking 10,000 families enrolled during pregnancy and followed over the first 5 years of the child’s life*.* In addition to creating a major research platform, ORIGINS also provides the infrastructure for harmonized nested subprojects integrated with clinical and diagnostic services [21].

### Participants

Eligible women for this study were pregnant women consented into ORIGINS when they attended their first hospital prenatal visit or via telehealth, that is, at 20 to 22 weeks’ gestation. Participants considered for inclusion in the MMM study were (1) women at 18 to 28 weeks of pregnancy; (2) aged ≥18 years; (3) able to read, write, and understand English; and (4) able to access the internet. We aimed to recruit 25 participants per randomization arm (75 participants in all). Overall, there is limited population diversity within the full ORIGINS cohort, who are predominantly White women, degree educated, and from moderately high socioeconomic backgrounds [21].

### Study Design

MMM is a pilot RCT with three parallel intervention groups: (1) MT, (2) LKCT, and (3) PMRT. The study used a mixed methods triangulation design that included both quantitative and qualitative data collected for our study outcomes. In combination, quantitative and qualitative methods complement each other and allow for a more robust analysis [22]. The qualitative data were collected both concurrently to the collection of quantitative data and secondarily to enrich the quantitative results.

### Procedures

Participants who undertook an initial clinic visit as part of standard ORIGINS protocols were provided with information about MMM by a research assistant; if they expressed interest, they were emailed links to a website and recruitment video [23]. Interested participants could then complete an e-consent form and web-based screening measures via REDCap (Research Electronic Data Capture; Vanderbilt University). In addition to assessing eligibility criteria, the screening assessment included the Edinburgh Postnatal Depression Scale (EPDS) [24]. If participants were above the threshold for the risk of depression (total score ≥13) or reported thoughts of self-harm (score of 1-3 on item 10), they received a telephone call from a member of the project team to discuss more targeted support services. They were not excluded from participation in the study.

Once consented, the participants were stratified by parity and randomized to 1 of the 3 intervention arms. Each participant was provided their web-based intervention condition using Teachable (version 1.0; Teachable, Inc), a web-based learning management system. Over 8 weeks, participants were given access to a weekly psychoeducation module (15-20 min), a daily meditation practice (7-15 min per day, with each meditation exercise repeated daily for 1 wk), and a weekly informal or *on-the-spot* practice that mapped to each theme. Each week, they were sent an automated reminder SMS text message and prompt to complete assessments, including an assessment of participation in the program as well as self-report measures of stress and emotion regulation. Participants who chose to opt out were directed to a survey to capture their reasons for opting out. Core themes and practices for each condition are described in Table 1. The training programs were available via the Teachable website and are minimal interventions with no direct interaction with a therapist or other users. The full study protocol includes detailed information on the battery assessment questionnaires administered before, during, and after the intervention, as well as the intervention format and structures [4].

**Table 1:** Outline of key themes and practices for each condition.

Components	Mindfulness training	Loving-kindness and compassion training	Progressive muscle relaxation training
Themes	Nonjudgmental awareness of present-moment experiences (thoughts, feelings, and sensations) Acceptance of present-moment experiences (thoughts, feelings, and sensations) Using the breath as an anchor for attention How mindfulness can support perinatal health	Friendliness toward self Loving-kindness toward self, baby, and others Self-compassion Responding to difficulties with kindness and compassion How loving-kindness and compassion can support perinatal health	Relaxation How relaxation can support perinatal health
Formal practices	Body scan, breath-focused meditation, sitting meditation, walking meditation, mindfulness of sound, and mountain meditation	Compassionate check-in, compassionate body scan, soothing image practice, loving-kindness for a loved one, loving-kindness for self, giving and receiving loving-kindness, and responding to emotions with kindness and compassion	Progressive muscle relaxation exercise
Informal practices	Mindfulness of daily activities (eg, chores, showering, and waking up) mindful communication, mindful walking in nature, and 3-minute breathing space	Body-softening practice, micromoments of soothing, on-the-spot loving-kindness practice, and savoring practice	Informal progressive muscle relaxation applied in different contexts (eg, going to bed, driving, and waking up)

Participants in the study were notified by email that they may be contacted for an interview once they had completed the intervention, regardless of how much of the intervention they completed. The qualitative interview data were collected through purposive sampling from all participants (n=84) after the intervention to enrich our understanding of engagement in the web-based training programs. The interviewer was blinded to the intervention arm of each participant.

### Data Collection (Quantitative and Qualitative)

#### Quantitative Measures

Participants’ involvement was measured through their regular engagement web-based survey, developed using REDCap by the first author in consultation with the project team. The survey included a range of categorical questions about the use of meditation and on-the-spot practices. The survey also included questions on barriers and enablers to exercises, and participants could select as many as appropriate from a given list. In addition, there was a free-text question for any other information they would like to share from their experience of the program in the preceding week (refer to Multimedia Appendix 2 for a copy of the survey).

A range of measures were collected in the full pilot RCT, as outlined in the protocol [4], but not analyzed and reported in this paper. Midway through the intervention, the project team assessed survey completion rates, and, owing to low compliance and perceived participant burden, the regular engagement survey collection frequencies were reduced from weekly to fortnightly.

#### Qualitative Measures

The regular engagement surveys included an opt-out question whereby participants could elect to discontinue their participation in MMM. If they did, they could indicate why (drop-down menu with “Other: specify” option) and provide free-text information for suggested program changes as well as any other comments.

Three months after the intervention, all participants were invited to partake in an interview by telephone or videoconference. These interviews investigated perceived acceptance and confidence to practice meditation or relaxation as well as intention to sustain practice and sought to identify ongoing barriers and enablers to participation. The semistructured interviews included key questions with sample probes. The key question themes explored perceived skills development, observed benefits, adverse experiences, and confidence to practice (eg, enablers and barriers), as well as self-assessment on sustaining ongoing practice. Participants were asked about the usability of MMM and general feedback on well-being supports for perinatal women. The interview topic guide was developed in conjunction with the project team (refer to Multimedia Appendix 3 for a copy of the topic guide).

All interviews were conducted by the first author (JAD) and transcribed verbatim, with interim feedback provided to the project team to review core themes arising from the interviews. Interviews ceased in the absence of general new themes and once the project team agreed that data saturation had been reached.

### Data Analytical Approach

Data were described using frequencies and percentages for categorical variables and means and SDs for continuous variables. Qualitative data were analyzed by the first author (JAD) using thematic analysis to code and identify emerging themes in an inductive manner, identifying the themes and reviewing and defining them [25]. A second researcher familiar with the thematic analysis process reviewed the primary analyst’s interpretation of the data, and codes and themes were decided after discussions between the 2 reviewers. Similar concepts were identified and collapsed into categories. Data were analyzed using a phenomenological approach (ie, as a description of experiences as consciously experienced by that person), and narrative themes were deduced until saturation point. Exemplar quotes that captured the essence of each category were included. Both sets of qualitative data were analyzed using qualitative content analysis [26], with a code assigned to each concept using NVivo (release 1.5; Lumivero) to facilitate data management and analysis.

## Results

### Participants

**Figure 1:**
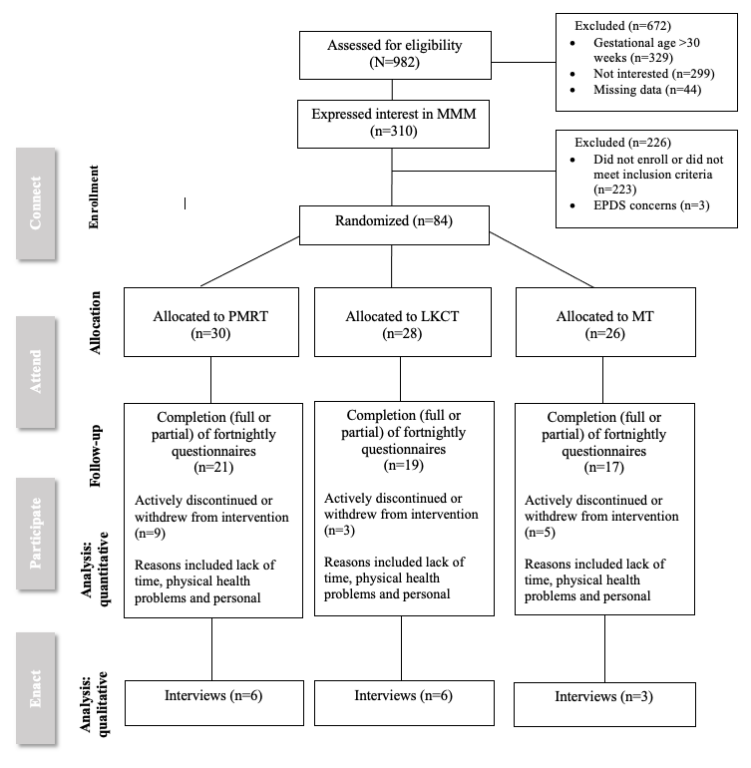
CONSORT Diagram.

In total, 982 pregnant women were exposed to this study while it was operational (approximately 60 new ORIGINS participants per month) from November 2020 to February 2022. Of the 982 participants, 310 (31.6%) expressed interest, and 84 (8.6%) consented and were randomized to 1 of the 3 intervention arms (Figure 1).

### Data Triangulation (Quantitative and Qualitative Data)

#### Overview

**Figure 2:**
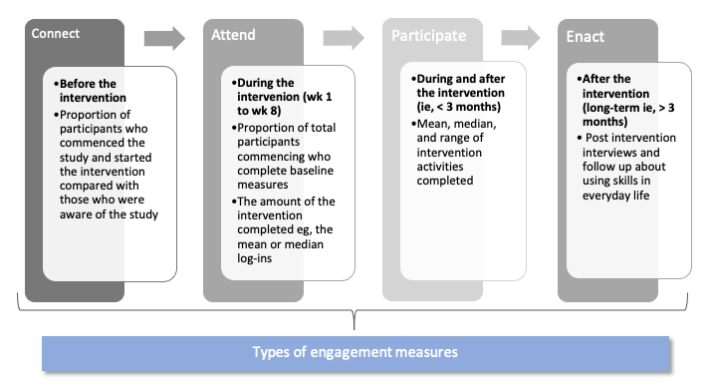
The connect, attend, participate, enact (CAPE) model.

Our quantitative and qualitative results are reported in combination in a sequential order following the CAPE framework. The totality of the quantitative and qualitative data was triangulated and interpreted together. Figure 2 indicates the CAPE measures that are reported in this study for participants’ engagement in the MMM program.

#### Connect: Exposure and Enrollment

*Connect* is defined as the proportion of participants who entered the study and started the DMHI compared with those who were aware of the study [19]. As this study was nested within ORIGINS, data were available on how many women were actively exposed to the study. A total of 982 pregnant women attended their first prenatal appointment over the 16 months that this study was operational (approximately 60 new ORIGINS participants per month) from November 2020 to February 2022. First, we explored the construct of *connect*, that is, 31.2% (310/982) of the participants were eligible and interested from the available cohort. Of the 310 women, 84 (27.1% [*connect* rate]) enrolled to participate in MMM. Of the remaining 226 women who did not proceed to randomization, 223 (98.7%) failed to complete the baseline surveys and timed out of eligibility (after 30 weeks’ gestation), and 3 (1.3%) displayed high psychological distress scores. The participants who were randomized in MMM (n=84) had a mean age of 32.7 (SD 4.0) years. The majority (65/84, 77%) identified as Australian, British, or European; 67% (56/84) of the participants fell in the advantaged or most advantaged socioeconomic status quintiles, with only 8% (7/84) falling in the disadvantaged or most disadvantaged quintiles [27]. Of the 84 women, 50 (60%) were university educated, with equal distribution across the 3 intervention arms (refer to Table 2 for participant demographics).

**Table 2:** Mums Minds Matter (MMM) participant demographics.

Characteristic			MMM group 1: PMRT^a^ (n=30)		MMM group 2: LKCT^b^ (n=28)		MMM group 3: MT^c^ (n=26)		Total (n=84)
Age (years), mean (SD)			33.2 (3.3)		32.7 (4.0)		32.1 (4.6)		32.7 (4.0)
Gestation (weeks), mean (SD)			25.6 (3.1)		25.0 (3.1)		25.7 (3.0)		25.4 (3.0)
**Ethnicity and ancestry, n (%)**									
	African	2 (7)		0 (0)		0 (0)		2 (2)	
	Asian	3 (10)		3 (11)		0 (0)		6 (7)	
	Australian	6 (20)		9 (36)		9 (35)		24 (30)	
	British	10 (33)		9 (36)		7 (27)		26 (31)	
	European (mainland)	4 (13)		2 (7)		9 (35)		15 (18)	
	Irish	3 (10)		1 (4)		1 (4)		5 (6)	
	New Zealand	1 (3)		1 (4)		0 (0)		2 (2)	
	Missing	1 (3)		3 (11)		0 (0)		4 (5)	
**Socioeconomic status: IRSAD^d^ quintiles, n (%)**									
	1: most disadvantaged or lack of advantage	1 (3)		0 (0)		0 (0)		1 (1)	
	2	2 (7)		2 (7)		2 (8)		6 (7)	
	3	10 (33)		4 (14)		7 (27)		21 (25)	
	4	12 (40)		11 (39)		8 (31)		31 (37)	
	5: least disadvantaged or most advantaged	5 (17)		11 (39)		9 (35)		25 (30)	
**Education of mother, n (%)**									
	High school	4 (13)		6 (21)		7 (27)		17 (20)	
	Trade	5 (17)		3 (11)		1 (4)		9 (11)	
	University	19 (63)		15 (54)		16 (62)		50 (60)	
	Other	2 (7)		2 (7)		2 (8)		6 (7)	
	Missing	0 (0)		2 (7)		0 (0)		2 (2)	

^a^PMRT: progressive muscle relaxation training.

^b^LKCT: loving-kindness and compassion training.

^c^MT: mindfulness training.

^d^IRSAD: Index of Relative Socio-Economic Advantage and Disadvantage. This index summarizes information about the economic and social conditions of people and households within an area, including both relative advantage and disadvantage measures [28].

As part of the screening process, participants completed the EPDS. Of the 84 women, 36 (43%) showed no indicators of depression, whereas 37 (44%) had mild depression scores, and 9 (11%) had moderate depression scores. Only 2 (2%) of the 84 participants scored in the severe range. All participants scoring above the threshold for the risk of depression (total score ≥13) received a telephone call from a registered psychologist to discuss referral to more targeted support services. They were not excluded from participation in the study.

#### Attend: Retention and Presence

*Attend* for digital interventions is a measure of the amount of the intervention completed and presence in the intervention, such as the number of participants who completed the intervention modules [19]. In this study, the *attend* dimension was measured using the percentage of participants who did not actively opt out of the study and of those who completed the regular engagement surveys in response to SMS text messages, including barriers and enablers. In total, across all program groups, 20% (17/84) of the participants actively opted out once they had started, consistently distributed across the groups. The main reasons for opting out of the study were as follows: not enough time (11/17, 65%), other (3/17, 15%), personal (1/17, 7%), concerns about health (1/17, 7%), and did not enjoy the program (1/17, 7%). Of those who provided feedback, the responses indicated that the women frequently felt too busy, and they suggested different ways, such as sending reminders, to help them overcome this issue:

My own schedule has prevented me from being able to use the audio sessions effectively. Maybe setting up a daily prompt at a set time convenient to each mum would assist one to remember.

We were limited in our ability to report the completion of weekly intervention modules owing to challenges accessing data from the Teachable platform; therefore, we are unable to conclusively report on those who had a *continuous presence* in the intervention every week. There were no notable differences among the intervention arms (refer to Table S1 in Multimedia Appendix 4, which indicates the proportion of those who were randomized who participated in the regular engagement surveys by intervention arm).

We were interested in understanding the barriers and enablers to ongoing study attendance. These are reported, respectively, in Table 3 and Table 4. Participants could indicate >1 barrier or enabler each fortnight, but not all participants completed this activity at each time point.

**Table 3:** Barriers (participants responded to the following question: “Did any of the following prevent you from doing the exercises this week?”).

Barrier (survey option)	Week 2: participants who responded (n=37), n (%)	Week 4: participants who responded (n=36), n (%)	Week 6: participants who responded (n=30), n (%)	Participants who responded at at least 1 time point (n=55), n (%)
“I didn’t have enough time”	13 (35)	11 (31)	9 (30)	25 (45)
“I got interrupted”	6 (16)	4 (11)	6 (20)	14 (25)
“I didn’t make it a priority”	9 (24)	13 (36)	13 (43)	27 (49)
“I didn’t have enough support or encouragement”	0 (0)	0 (0)	1 (3)	1 (2)
“I forgot”	15 (41)	12 (33)	14 (47)	27 (49)
“Other”	5 (14)	4 (11)	4 (13)	10 (18)

**Table 4:** Enablers (participants responded to the following question: “Did any of the following help you to do the exercises this week?”).

Enabler (survey option)	Week 2: participants who responded (n=37), n (%)	Week 4: participants who responded (n=36), n (%)	Week 6: participants who responded (n=30), n (%)	Participants who responded at at least 1 time point (n=55), n (%)
“Setting aside dedicated time each day”	12 (32)	10 (28)	9 (30)	19 (35)
“Ensuring I had a quiet space to do the exercise”	12 (32.4)	14 (39)	12 (40)	27 (49)
“Scheduling it at a specific time of the day”	5 (14)	8 (22)	5 (17)	12 (22)
“Having support and encouragement from someone”	2 (5)	2 (6)	1 (3)	5 (9)
“Other”	5 (14)	4 (11)	4 (13)	11 (20)

The fortnightly surveys enabled us to capture general comments as the participants progressed through the program, although response rates were low across all groups. There were observed differences in general comments among the groups, but these can only be interpreted as illustrative (Textbox 1; for all comments by theme, refer to Table S2 in Multimedia Appendix 4).

**General weekly comments of those who responded.**

- For Mums Minds Matter (MMM) group 1 (progressive muscle relaxation training [PMRT]), the most frequent theme was that participants were too busy, had other commitments, and found the program an additional duty:
  “While I would like to continue, I do not see me finding the time with my child and worry that I will find I have more issues...Not being able to find the time has also added to my stress because I felt like I was failing.”
- Others in group 1 found the tool helpful in their daily activities:
  “It’s been helpful and doing the meditation at night, I’ve remembered during the day to stop and take some deep breaths.”
- Fortnightly comments from participants in MMM group 2 (loving-kindness and compassion training [LKCT]) included the perception of the program as a “treat”:
  “This was my first week, and so far, I have absolutely loved it. Just learning to check in and treat myself like a friend and be conscious of the way I talk to myself in my head with compassion rather than judging or being super critical or worrying about others being critical.”
- Other feedback indicated that it was difficult to concentrate on the program:
  “Sometimes I find it very difficult to focus on the program and my mind wanders, it would help for more strategies for this.”
- Participants in MMM group 3 (mindfulness training [MT]) provided a number of comments on program dislikes:
  “I found it hard to be in the moment when walking. I was a lot more distracted.”
  “I find the program a bit repetitive doing the same exercise every day.”

### Participation and Enactment: Sustained Practice

#### Overview

*Participation* is operationally defined as the completion of intervention activities, that is, *active engagement* with the intervention material [19]. *Enact* refers to the participant making use of intervention strategies or knowledge as part of their daily life [20]. In this study, we assessed active engagement and ongoing skills use through postintervention interviews. We undertook a total of 15 interviews with participants, conducted 1 month to 3 months after the intervention (MMM group 1 [PMRT]: n=6, 40%; MMM group 2 [LKCT]: n=6, 40%; and MMM group 3 [MT]: n=3, 20%). The interviewer was blinded to the type of intervention to which participants had been randomized. We have reported both aspects of participation and short-term enactment together with higher and lower order themes deduced from interview data. Our primary objectives were to investigate perceived acceptance and confidence to practice meditation or relaxation as well as intention to sustain practice and to identify ongoing barriers and enablers to participation. The data were examined both without knowing group or intervention assignment and again with this knowledge. As there were no consistent patterns of responses in the data across these groups, themes were constructed based on the data as analyzed together. We reached inductive thematic saturation as described by Saunders et al [29] across the program arms and did not stratify and report the themes by program type because there were no major thematic differences.

**Figure 3:**
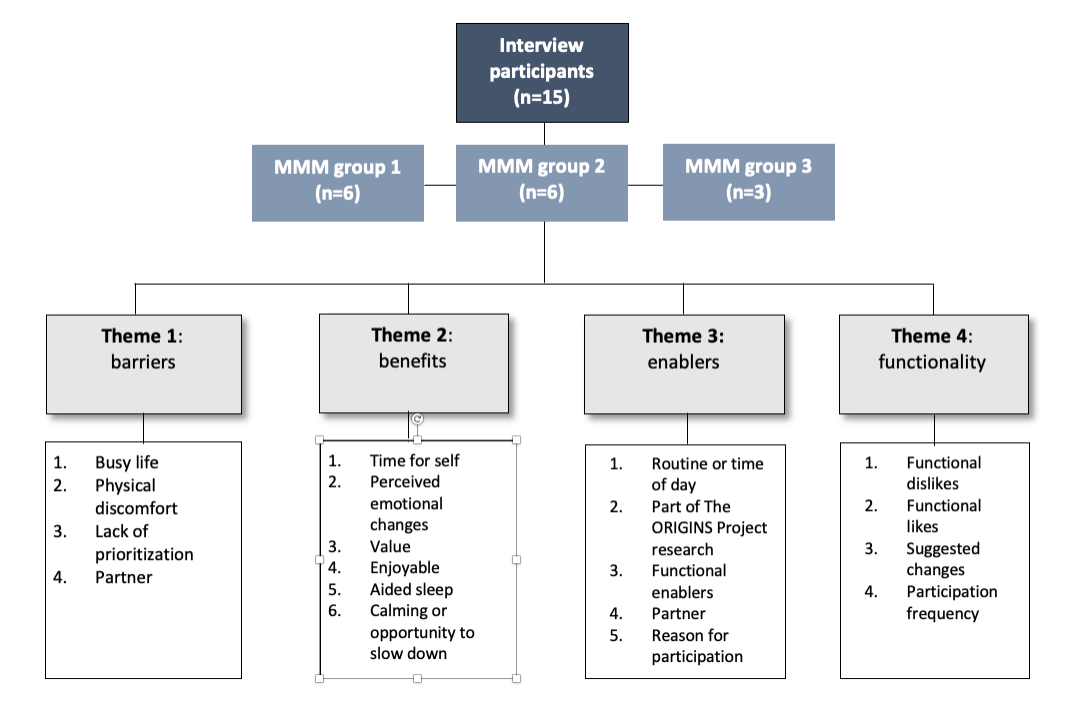
Key interview themes. MMM: Mums Minds Matter.

Key interview themes are presented in Figure 3 and in a thematic tree map by frequency of coding references (Multimedia Appendix 5).

#### Theme 1: Barriers

Overwhelmingly, the predominant barrier to undertaking a web-based training program reported by participants in this study is a busy life. Physical challenges, especially those associated with pregnancy, created barriers, such as simply being too tired, general discomfort, and physical health problems such as back pain:

Getting to the end of the day and kind of going I should do it before I go to bed...and then you’re kind of like, “No. I’m just shattered,” and going to bed instead.

MMM-24: MT

Many participants perceived the potential benefits but simply did not prioritize the program or felt a sense of duty to participate:

It all became about, yeah, my priority level of trying to carve out time for me to do it, versus everything else going on.

MMM-22: MT

Like it was fun for the first, like, couple of weeks, then I’m just like—I don’t know, it’s just not for me.

MMM-27: LKCT

In particular, the sense of duty was expressed in relation to being part of ORIGINS and an intrinsic sense of needing or wanting to carry out the research requirements. This can be a motivator for some but for others it created a sense of failure:

[I’m] almost doing it for you guys rather than doing it for myself.

MMM-31: PMRT

A partner can be both a barrier and enabler: some partners were encouraging, whereas a few participants expressed concerns about taking time away from their partners:

The only thing is that when I told my husband that, “Sorry, I’m away for 10 minutes,” sometimes it wasn’t the best part of the day for him to be left alone.

MMM-38: LKCT

#### Theme 2: Benefits

Unsurprisingly, participants acknowledged the importance of taking time out for themselves. This was frequently reported as a key benefit of an emotional well-being program:

So, I think using this, just to give myself that half an hour just for me, and actually be okay with it, being a little bit more selfish for myself as well, I think, really helped me stick to it.

MMM-32: LKCT

I think because it’s never a bad thing to take time for yourself and especially when life can get so busy, and, you know, stopping to take a few deep breaths is never going to be a bad thing.

MMM-36: LKCT

Participants reported emotional changes and variations in mindset as a benefit of undertaking the program:

I’d say it probably helped me acknowledge that I have been struggling with things because it asked that question.

MMM-25: PMRT

There was recognition of the value in the program, and many found it enjoyable:

I think mental health is really, really important, and so anything to check in with myself but also contribute to efforts working towards that is really valuable.

MMM-24: MT

So, at the beginning, it was every day. I absolutely adored it...I really think—especially the beginning, when I was very disconnected from myself, I think it really helped me get back on board with the positive side of being pregnant and not keeping my head in the past and all the issues that could go wrong with the pregnancy.

MMM-32: LKCT

The training also aided sleep, which is acknowledged as important to pregnant women:

Just before bed, it just was able to relax me so that I can have a better night’s sleep. Sometimes when you’re tossing, and turning so much, you can’t relax when you get into bed. So, it’s just helpful to help wind down at the end of a busy day.

MMM-08: MT

One of the predominant reasons in finding value was a sense of calmness that the training evoked and an opportunity to slow down:

So, it kept me calm throughout the—I think it’s 8 weeks...It kept me really calm with the way things were going in my life. So, that really helped me, 10 minutes a day was not hard for me, just sitting there and listening to it.

MMM-16: PMRT

I think dealing with your own mental health whether you have mental health problems before or not, here’s a good thing to look at before coming into, I guess, the biggest change of your life...Spending that time in your pregnancy, I guess, trying to learn some of those practices and just slow down life, you know, visualize, stop, relax, yeah, and learn those sort of coping mechanisms.

MMM-43: LKCT

#### Theme 3: Enablers

We were interested to learn what supports facilitate participation and engagement in a DMHI. Finding a regular time of day helped many women as did viewing it as similar or adjacent to regular exercise:

First thing in the morning...particularly if I’ve done some exercise, then it’s a really good way to cool down from the exercise and also get ready for the day. I found that’s actually been the nicest time to do it.

MMM-24: MT

It was setting the time to do it every day and having the routine of it.

MMM-43: LKCT

Being part of a wider research study acted as an enabler, as reported by several women:

I just think it’s really important to help with studies, so I’m happy to put my hand up for, you know, studies, especially being on mat. [maternity] leave. You know I’m not at work anymore; so, it’s good to have other things to feel that you’ve been productive with.

MMM-33: MT

I probably wouldn’t have even looked into it if I wasn’t doing The ORIGINS Project.

MMM-27: LKCT

Many participants discussed a range of functional enablers, such as the importance of regular reminders via SMS text message or email and the fact that the training app needs to be accessible and easy to use on different devices:

It needs to be easy, quick, you know, you don’t want to be logging in and out of an app all the time ‘cause often, obviously as a mum, you don’t have time to fiddle around with passwords and logging on and off.

MMM-33: MT

The perception that it was a useful tool was also acknowledged:

[J]ust to give myself a bit of a focus every day.

MMM-32: LKCT

In addition, it was convenient:

I think what helped was basically if I didn’t necessarily have to make any changes to my routine.

MMM-20: MT

Predominantly, partners were perceived as supportive of the training, and a few participants reported undertaking the modules together:

I think there are some nights where I’m a bit tired, he’d be like, “No, let’s do it.”...So, it’s like going to the gym and having a gym buddy, somebody else to encourage [you] to do it.

MMM-08: MT

Participants discussed their motivation for participation, with several citing a personal or family history of mental illness and mental health problems, including bipolar disorder, postnatal depression, and anxiety:

I was more anxious in the beginning of the pregnancy, obviously, not knowing what I’m going to be expecting down the track. So, I was quite nervous and anxious at that point in time, but then with this program, it’s actually calmed me down, it’s made me peaceful.

MMM-16: PMRT

#### Theme 4: Functionality

Participants disclosed a range of functional dislikes about the web-based training. These included the trainer’s voice (“annoying” and “too Australian”), the repetitiveness and similarity of the modules, and the frequency of daily practice:

It’s quite long. I think maybe trying to do it every single day got a bit much.

MMM-27 LKCT

A common theme was the dislike of the walking mindfulness practice, which was an exercise in the MT condition:

About week 3, it became a walking mindfulness, and I found that’s a lot harder to incorporate into life.

MMM-20: MT

There was a lot of like walking...and if I’m going to do a meditation, I want it to be me lying down and chilling out.

MMM-33: MT

The web-based platform itself was difficult to use, requiring participants to log on to a website, which some found frustrating and not “user friendly.”

Participants also reported many functional likes about the web-based training, with some perceiving it as a “treat”:

I think it was always a real treat anytime I’d have to do it.

MMM-20: MT

Additional likes included the tone, the pace, the music, and the brevity:

Ten minutes a day was not hard for me, just sitting there and listening to it.

MMM-16: PMRT

Interview feedback provided a breadth of suggested changes to the programs as well as other preferences, including human contact, such as telephone calls or group sessions:

Physical sessions would be good, and it might help mums connect with other mums in the same situation.

MMM-13: LKCT

In terms of program changes, suggestions included the ability to choose topics, daily reminders, inclusion of case studies, a calendar function, and more variety, such as diversity in sounds:

Different sounds, like sometimes water, sometimes forest...even different voices sometimes.

MMM-31: PMRT

Links to additional information or further reading were also suggested.

Repeatedly, participants described initial enthusiasm for the training that waned as the weeks progressed. There was a clear sense of personal pressure to perform the exercises, and often more realistic goals were set:

The goal would be to two to three times a week. I found that that seems to be enough to maintain but also not feeling too pressured that I have to get it done.

MMM-24: MT

Others maintained regular daily practices*:*

So, we just set a time at 9 o’clock, we’d stopped doing whatever we’re doing, go to bed, so devote that 15 minutes beforehand for meditation.

MMM-08: MT

## Discussion

### DMHI Engagement Using the CAPE Model

The overall aim of this study was to characterize participant engagement and experience in a prenatal digital well-being pilot program using an adaptation of an existing participant engagement model (CAPE). Our results provide insights into the barriers and enablers to participation as well as functional enhancements, including variety in sounds and content, but not necessarily notable differences in engagement by the type of intervention. These findings are useful contributions to understanding participant experience and engagement to adapt for a full trial.

First, we explored the construct of *connect*, that is, the proportion of participants who were eligible and interested from the available cohort (310/982, 31.6%) and those who were randomized to start the intervention (84/310, 27.1%). Across all program groups, 20% (17/84) of the participants *actively* opted out, although more participants may have disengaged from the intervention but did not actively withdraw. The predominant reasons for withdrawal were *busy life* and *other priorities*. There seems to be a paucity of literature reporting on the available population of individuals who are exposed to digital well-being interventions and who then enroll and continue in the training programs [19]. This study was promoted to an existing cohort population who may be more motivated to participate but who may equally be overexposed and fatigued by their participation in research.

Researchers should consider issues related to participants’ motivations for engaging in research [30] compared with motivating factors for engaging in interventions because these could be distinctly different and will affect real-world implementation. During the course of pregnancy, women may experience a variety of psychological changes, including developing the motivation to change their lifestyle habits [30]. Thus, pregnant women are likely to be motivated to undertake behavior change, making them an ideal target group for interventions. A recent scoping review exploring behavior change in pregnant women [31] highlighted the role of behavior change programs in pregnancy that empower women and which may be beneficial throughout their lives. Understanding why and how women want to change will ensure that future health promotion efforts are appropriately tailored.

Second, our results reported on *attend,* that is, the amount of the intervention completed and presence in the programs, also referred to as “stickiness” [32]. One of the disadvantages of digital interventions is the lack of “stickiness” compared with other modes of contact, leading to dropouts from the study or a reduction in the intervention effect [33]. Capturing para-data, that is, the process by which the data are collected, provides an indication of the breadth and depth of participant use of web-based interventions [33]. Although this information can be used to interpret continuous presence in an intervention, it is not nuanced sufficiently to elicit why participants drop out and what might keep them engaged for longer periods or to program completion. For many digital health trials, particularly those conducted on the internet and particularly with self-help applications, high dropout rates may be a natural and typical feature [34], but it is still important to understand participants’ reasons for dropping out.

In this study, we were limited in our ability to interpret intervention compliance but were able to report on those who actively elected to *opt out* and why. The time required to complete the program was the predominant factor for opting out and cited frequently as a weekly barrier to participation. This information is unsurprising, considering the target audience of pregnant women and new mothers, and informs the design of future apps, whereby programs need to balance perceived time constraints against the fact that *having time to slow down and self-reflect* is likely one of the active ingredients of the program. In terms of enablers, setting aside dedicated time or space facilitated increased program engagement, which we would actively promote in future program design.

Third, we explored *participation* and *enactment,* that is, the completion of intervention activities and the application of intervention strategies in daily life. Our results yielded additional information about participant barriers and enablers, the benefits of the programs, and suggestions to enhancing functionality, including the ability to choose topics, daily reminders, the inclusion of case studies, a calendar function, and more variety and diversity in background sounds. Participants struggled with the physical challenges associated with pregnancy, such as back pain, and some commented on their dislike of performing walking mindfulness exercises while pregnant. A systematic review and meta-analysis of MBIs suggests that, among healthy perinatal populations, MBIs had smaller effects than those found from MBIs in other health populations [35]. Our results suggest that this might be in part because changes related to pregnancy (eg, muscle soreness and increased fatigue) might interfere with willingness or ability to engage in these interventions. More robust investigation, enhancing practice and investigating intervention engagement, is needed [35] to design appropriately targeted strategies to optimize participation in perinatal well-being programs.

### Future DMHI Design Approaches

In general, women report that time is the prevailing issue, and although they understand the potential benefits of the intervention, in reality, their lives already feel too busy. Future intervention design approaches should consider making an intervention as minimal and as engaging as possible to achieve the desired result (ie, decrease time commitment). In our previous study, pregnant women indicated their preferences for these types of interventions of <4 modules per week, <15 minutes in duration, delivered over a 4-week period [36]. In addition, future consideration should be given to women being provided guidance by health care providers to find prioritized or dedicated time as part of their prenatal self-care. Furthermore, personalization and technological advances are likely to optimize the impact of well-being interventions [6].

Evidence suggests that meditation and related contemplative practices decrease stress levels and increase mindfulness, often accompanied by the experience of inner calmness, self-worth, and self-respect, as well as promote well-being [37]. A meta-analysis of web-based mindfulness-based stress reduction and other MBIs demonstrated their potential to contribute to improving mental health outcomes, particularly stress [38]. Taking time out for themselves was a vital motivator for many study participants, who reported finding value in the sense of calmness that the training provided and the opportunity to focus and slow down. It is not necessarily clear whether the type of contemplative practice makes a difference, although there is some evidence of the superior effects of LKCT over MT [39,40] but not necessarily in pregnant women. Our study underscores that the opportunity for reflection and an MBI are generally perceived positively. This finding complements a recent meta-analysis of web-based MBIs [41], which demonstrated that a range of web-based MBIs (in which there is a boom) are beneficial for improving mental health outcomes in many populations. It is recommended that future research should follow up with participants to elucidate longer-term enactment and sustained well-being practices.

Understanding the pragmatic use and functionality of digital training programs will contribute to optimal design strategies. Our results indicated that participants want brief, easily accessible, and tailored web-based training programs, with frequent reminders or *nudges*. Some participants indicated that they would like more interaction and direct contact with trainers or peers. This is in line with research predicting engagement with a digital psychosocial intervention for psychosis [42] based on coaching, such as asynchronous email support, that promotes engagement; however, this was in a treatment cohort who were motivated to recover.

Overall, our findings contribute to the rationale that DMHIs need to be tailored to the type of population targeted. Although a recent systematic review provided preliminary evidence that web-based interventions can be a promising and advisable form of intervention during the prenatal period [16], there is a paucity of evidence regarding the long-term effectiveness of these programs [43]. Further research should consider different design options to strengthen engagement, including usability, a variety of features and options, personalization, credibility, informative content, and user control. Providing features that allow users to customize an app to suit their individual needs and preferences is a good way to improve user satisfaction as well as user experience and consequently reduce attrition rates [44].

### Unknown Impacts of the COVID-19 Pandemic

Finally, consideration should be given to the unknown impacts of the COVID-19 pandemic during this time. Throughout this study, there were periods of enforced lockdown restrictions, both locally and globally. The large-scale impacts of lockdown restrictions on social, emotional, and economic well-being are predicted to have unparalleled and extensive implications for mental health in general populations, independent of the biological effects of infection [45]. It has been indicated that positive mindsets may be protective against psychological distress for the mother and her child, suggesting that meditation-based or similar training might help support expectant and postbirth mothers during times of crisis, such as a pandemic [46]. In addition, the application and acceptability of digital communications have increased exponentially. Thus, externalizing factors on a macroscale may have increased participants’ motivation, compliance, and engagement in this study. The reliance on, and acceptance of, digital and internet-based interactions is likely to continue as entrenched and normalized behaviors change.

### Limitations, Strengths, and Future Research

This study has several limitations and strengths to consider. As already noted, participants in the study are part of a longitudinal research cohort and could be considered biased in their willingness to engage, although the direction of this bias is unclear. Nonetheless, the richness of the qualitative data provided opportunities to capture their motivations, with several participants confirming that they were undertaking the study to contribute to research rather than for personal benefit. A limitation of this study was our inability to access program use through the participants’ web use of the Teachable platform. These data were unavailable but could have provided extra insights into participant behaviors such as log-on duration, frequency, and time of day. In addition, there is limited population diversity within the ORIGINS cohort, who are predominantly White women, degree educated, and from moderately high socioeconomic backgrounds. This limits the generalizability of our findings, and we recommend undertaking future research with prenatal women from more diverse backgrounds.

In addition, our results, particularly the interview data, did not identify any major differences among the experiences of participants exposed to the 3 different intervention arms. The interview questions were centered on skills development, enablers and barriers to the intervention as well as its benefits, and confidence to practice. This meant that there was consistency in themes, irrespective of program allocation, and the project team reached data saturation after 15 interviews. Future research in this field should consider the inclusion of questions related to the type of practice to determine whether engagement is influenced by the practice itself.

Although the EPDS was administered as a screener, we did not exclude participants who had moderate or severe scores, although some self-selected to withdraw. Numbers were low in these categories, but we were able to compare the EPDS scores between those who discontinued with those who did not, and there was no significant difference. There was also no difference between those who did not complete any of the surveys and those who completed at least 1 survey. We did not explore any direct associations between moderate to severe depression and program engagement. It would be useful to factor this into future studies.

### Conclusions

In general, there are few studies of digital interventions that follow expectant mothers from pregnancy and through childbirth [43]. This may be particularly important in view of the fact that maternal psychological distress may occur before pregnancy, during pregnancy, and after childbirth, thereby making prevention and intervention throughout the entire perinatal period important [43]. Providing convenient and easy access to engaging and effective prevention programs is critical. Our research highlights the appeal and feasibility of intervention approaches based in contemplative practices for perinatal women delivered in a digital format. It contributes to the extant literature on targeted approaches to effectively engage women during pregnancy to support their mental well-being and to develop skills for long-term enactment. The data collected will be used to determine the feasibility of the study for full-scale trial in a broader population group with the ultimate goal of developing a universal health promotion tool with widespread reach and uptake.

## Appendix

Multimedia Appendix 1 Logic model.

Multimedia Appendix 2 Regular web-based participant check-in.

Multimedia Appendix 3 Interview topic guide.

Multimedia Appendix 4 Proportion of participants who were randomized who responded to the fortnightly SMS text message about their intervention experience, and general weekly comments of those who participated.

Multimedia Appendix 5 Thematic tree map.

## Abbreviations

CAPE: connect, attend, participate, enact
CBI: compassion-based intervention
DMHI: digital mental health intervention
EPDS: Edinburgh Postnatal Depression Scale
LKCT: loving-kindness and compassion training
MBI: mindfulness-based intervention
MMM: Mums Minds Matter
MT: mindfulness training
PMRT: progressive muscle relaxation training
RCT: randomized controlled trial
REDCap: Research Electronic Data Capture

## Data Availability

The nonidentifiable data presented in this study are available on reasonable request from the corresponding author.
