# Supplementary figures and images for: Perinatal Women’s Perspectives of, and Engagement in, Digital Emotional Well-Being Training: Mixed Methods Study
Source: J Med Internet Res. 2023 Oct 17;25:e46852. doi: 10.2196/46852 (PMC10618893; doi:10.2196/46852)

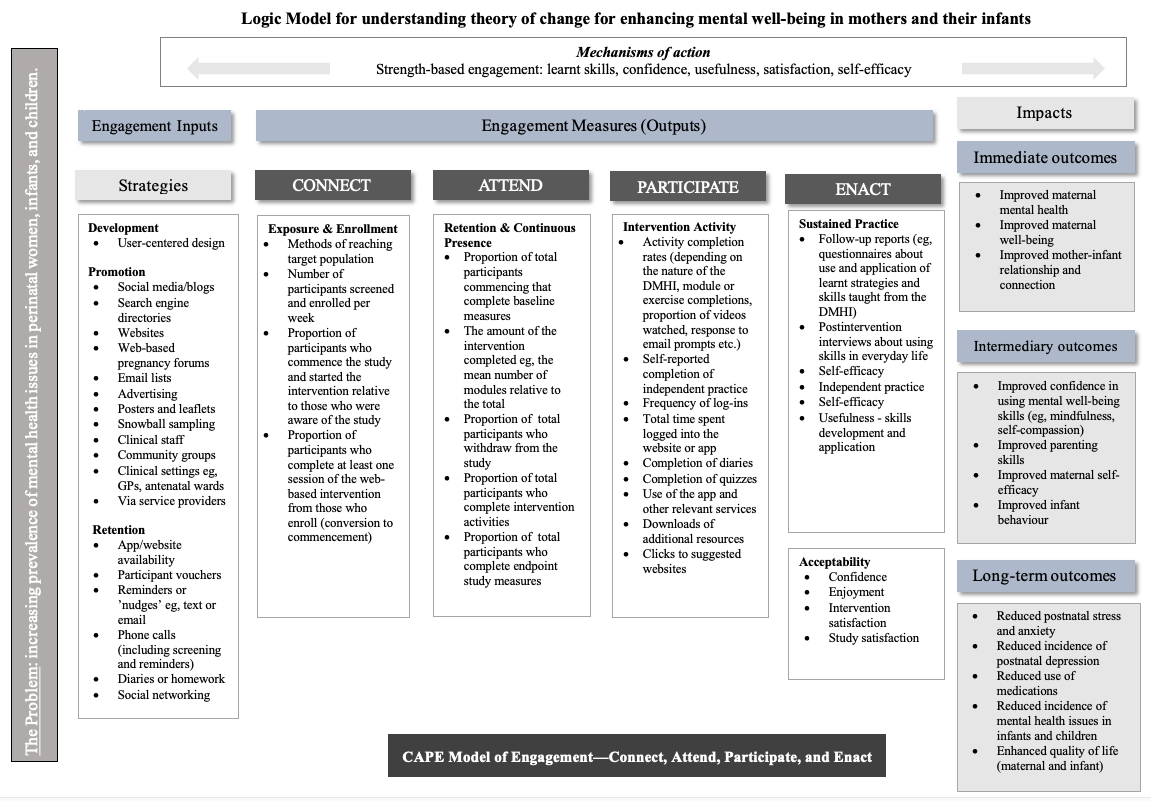

Supplement: Multimedia Appendix 1 [file jmir_v25i1e46852_app1.png]

### Thematic TreeMap (frequency of coding references)


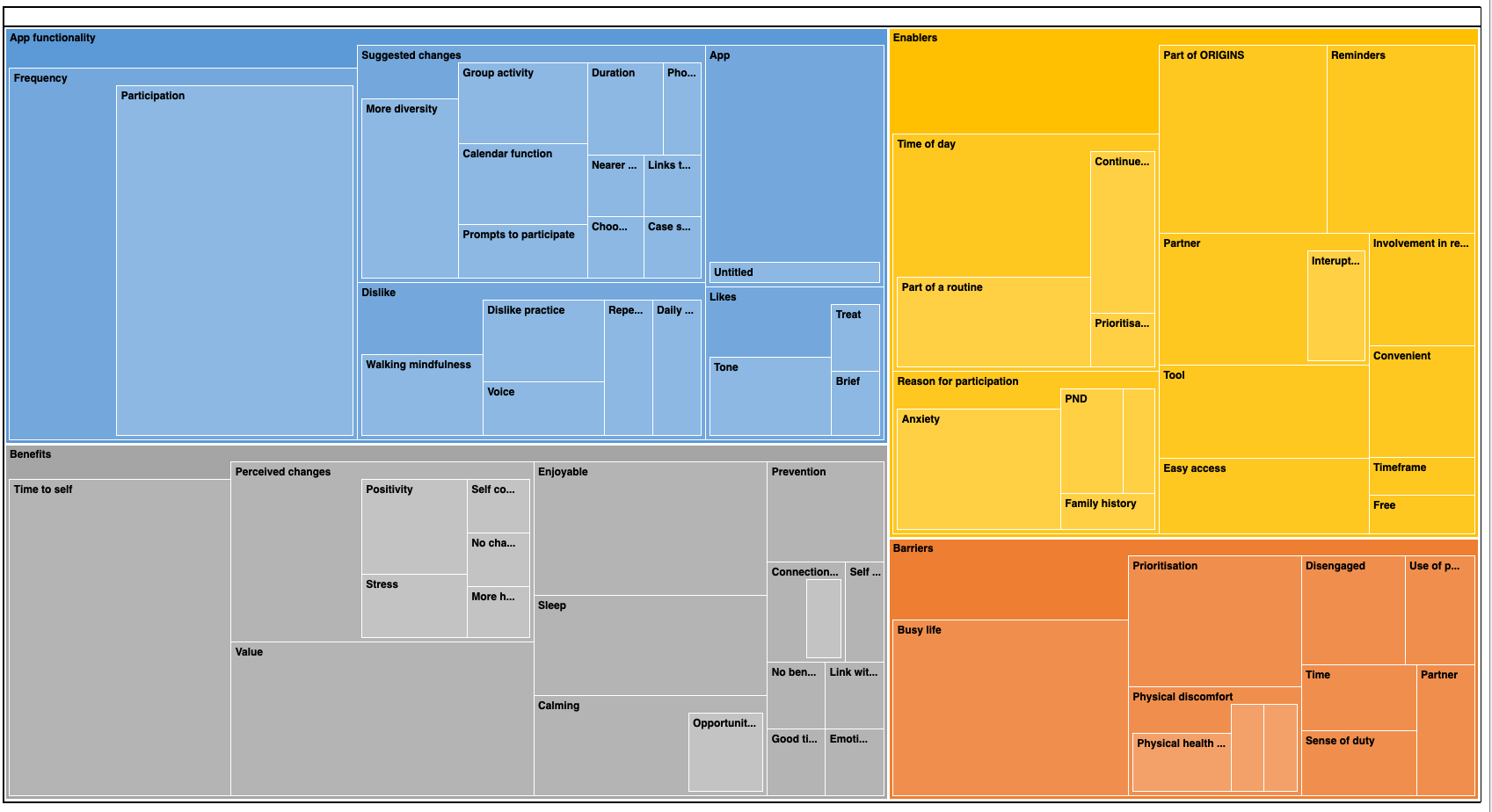

Supplement: Multimedia Appendix 5 [file jmir_v25i1e46852_app5.docx]
