# Supplementary material for: Perinatal Women’s Perspectives of, and Engagement in, Digital Emotional Well-Being Training: Mixed Methods Study
Source: J Med Internet Res. 2023 Oct 17;25:e46852. doi: 10.2196/46852 (PMC10618893; doi:10.2196/46852)
Supplement: Multimedia Appendix 2 [file jmir_v25i1e46852_app2.pdf]

Weekly Check

Welcome to your weekly Mums Minds Matter Check In. We want to know if you are able to undertake your weekly exercises and practice sessions. Here are four brief questions to understand your experience. We will check in with you each week to see how you are going. Please remember this is to support you and if you are unable to complete exercises or practice one week, we hope you will be able to pick them back up the following week.

Thanks for taking part in the Mums Minds Matter study! It's time for your weekly check-in.

Have you tried the audio exercises this week?

- ◯ Yes, every day this week
- ◯ Yes, on 4-5 days this week
- ◯ Yes, on a couple of days
- ◯ Yes, but only once this week
- ◯ No, I did not try them this week but intend to next week
- ◯ No, I did not try them this week and I'm unlikely to next week

---

Have you tried the on-the-spot practices this week?

- ◯ Yes, once
- ◯ Yes, more than once
- ◯ No

Did any of the following help you to do the exercises this week? (answer as many as appropriate):

- ☐ Setting aside dedicated time every day
- ☐ Ensuring I had a quiet space to do the exercise
- ☐ Scheduling it at a specific time of day
- ☐ Having support and encouragement from someone
- ☐ Other

Did any of the following prevent you from doing the exercises this week? (answer as many as appropriate):

- ☐ I didn't have enough time
- ☐ I got interrupted
- ☐ I didn't make it a priority
- ☐ I didn't have enough support or encouragement
- ☐ I forgot
- ☐ Other

Is there anything else you would like to tell us about the program this week?

____

Would you like to opt-out of the program and/or the study?

- ◯ Yes
- ◯ No

Thank you for taking part in the Mums Minds Matter Weekly Check In. Next week, would you prefer we contact you by the following:

- ◯ Text message
- ◯ Phone call
- ◯ Email

Powered by REDCap
