# Supplementary material for: Perinatal Women’s Perspectives of, and Engagement in, Digital Emotional Well-Being Training: Mixed Methods Study
Source: J Med Internet Res. 2023 Oct 17;25:e46852. doi: 10.2196/46852 (PMC10618893; doi:10.2196/46852)
Supplement: Multimedia Appendix 3 [file jmir_v25i1e46852_app3.docx]

## Mums Minds Matter: An Evaluation of the Engagement and Experience of Perinatal Women in Emotional Health & Wellbeing Training Programs

Good morning/afternoon,

Thank you for taking the time to speak to me today. Your experiences in participating in Mums Minds Matter are really important to us.

The aim of this study is to understand your experiences in the Mums Minds Matter intervention, both during the intervention and afterwards. The research team are looking for feedback from women who used the program – even if you only used it a little bit. We will use the information collected to improve the program.

Participant Interview Outline

Preamble:

Today we will be asking you a few questions about your experiences of participating in Mums Minds Matter. The research team is interested in your opinion on the usability of the program and what you learned, as well as anything you have continued to use. We are also interested in how the program can be improved.

This conversation will be recorded, and answers transcribed, so that general themes can be analysed. All data gathered through interviews will be de-identified and remain confidential.

Do you have any questions?

**Okay, let’s begin…**

Participant Topic Guide

**Research question:** *Do characteristics of antenatal engagement and experience in an online minimal contact intervention differ by the type of emotional wellbeing training (mindfulness, self-compassion or general relaxation)?*

**Overall aims**:

1. To characterise participant engagement and experience during and post intervention in an antenatal digital emotional wellbeing pilot program.
2. To determine engagement differences in the type of training provided (mindfulness versus self-compassion versus general relaxation).

| 1. **Aim**: Explore engagement and perceived skills development in the program | 1. **Aim:** Explore perceived benefits or adverse experiences associated with the program | 1. **Aim:** explore confidence to complete program and continue to practice | 1. **Aim:** explore usability of Mums Minds Matter | 1. **Aim:** gather suggestions about how to improve access to health and wellbeing support for pregnant women |
| --- | --- | --- | --- | --- |
| **Main question:**  How frequently did you engage with the MMM program? | **Main question:**  Do you feel you benefited from participating in the MMM program? If so, what benefits did you experience? | **Main question:**  Are you confident to continue to practice? | **Main question:**  How usable is the Mums Minds Matter program? | **Main question:**  In your view, how can the wellbeing needs of pregnant women best be supported during and post-pregnancy? |
| **Sample probes:**   - How frequently did you use the program? - What helped you to use it? What got in the way? - How could the program be more engaging? If *yes*, it what ways? - What prompted you to participate in MMM? - Overall, did you enjoy your participation in MMM? - If *yes*, why? - If *no*, why not? | **Sample probes:**   - Do you feel your general emotional wellbeing is different since commencing MMM? - If *yes*, it what ways? - If *no*, why not? - Did you experience any adverse effects? - If so, what were they? | **Sample probes:**   - Will you complete the program? - Do you think you will continue to undertake regular wellbeing practices? - If *yes*, can you please tell me more about this [frequency, time of day, setting etc]. - If *no*, why do you think you haven’t? | **Sample probes:**   - What helped you do the exercises eg setting aside dedicated time each day? - What prevented you completing the exercise eg didn’t make it a priority? - Are there any other features you think could be added to the MMM program? - Would you recommend the MMM program to other pregnant women? - If yes, why? - If no, why not? | **Sample probes:**   - Are there other types of emotional wellbeing practices that you undertook while pregnant? - Do you have any other general comments about the program? |

**Interview close.**

We have come to the end of the interview. I don’t have any more questions for you, but do you have anything else you would like to say about what we have discussed today?

We will be undertaking a further interview of MMM participants in 12-months’ time. Would you be interested in participating?

[Yes / No / Unsure]

Thank you for your time today, it has been really valuable to talk with you. Would you like a copy of the final research paper?

**Enjoy the rest of your day.**
