# Supplementary material for: Perinatal Women’s Perspectives of, and Engagement in, Digital Emotional Well-Being Training: Mixed Methods Study
Source: J Med Internet Res. 2023 Oct 17;25:e46852. doi: 10.2196/46852 (PMC10618893; doi:10.2196/46852)
Supplement: Multimedia Appendix 4 [file jmir_v25i1e46852_app4.docx]

## Multimedia Appendix 4

###

### Table S1. Proportion of those who were randomised who responded to the fortnightly text about their intervention experience.

| **Week** | **MMM Group 1**  **PMR** | **MMM Group 2**  **LKCT** | **MMM Group 3**  **MT** | **Total** |
| --- | --- | --- | --- | --- |
|  | **(*n* = 30)** | **(*n* = 28)** | **(*n* = 26)** | **(*n* = 84)** |
| **2** | 10 (33.3%) | 14 (50.0%) | 13 (50.0%) | 37 (44.0%) |
| **4** | 14 (46.7%) | 11 (39.3%) | 11 (42.3%) | 36 (42.9%) |
| **6** | 10 (33.3%) | 15 (53.6%) | 5 (19.2%) | 30 (35.7%) |
| **Post Test** | 8 (26.7%) | 7 (25.0%) | 7 (26.9%) | 22 (26.2%) |

## Table S2. General weekly comments of those who participated.

| **Group** | **Theme** | **Examples** |
| --- | --- | --- |
| **MMM1:**  12 participants in total responded | Too busy and other commitments | If I was working all day, I was less likely to do it as once I got home and prepared dinner, kids lunches, etc I was either tired or forgot. One day I tried at bedtime I fell asleep. |
|  |  | A particularly busy week for work and other commitments, this week I loved the activity and wanted to do it but just found it hard to squeeze in. |
|  |  | Working full time (5 days a week) a husband doing shift work, and 2 children under 5 makes it really hard to find time before bed to do the exercises. |
|  |  | While I would like to continue, I do not see me finding the time with my child and worry that I will find I have more issues… Not being able to find the time has also added to my stress because I felt like I was failing. |
|  | Helpful tool | It's been helpful and doing the meditation at night, I've remembered during the day to stop and take some deep breaths. |
|  | Triggering | Additionally, I do not find mindfulness a practical tool for someone with dissociation like myself as it triggers further dissociation. Mindfulness can be a great technique but for some disorders it is just not beneficial. |
|  | Too repetitive | I don’t feel doing the same exercise everyday helps, it’s good to have a different track daily |
|  | Functionality | Unfortunately the app and the website have not been working, thus I have been unable to complete the task. I have emailed notifying there have been some issues. |
| **MMM2**  10 participants in total responded | Treat | This was my first week, and so far I have absolutely loved it. Just learning to check in and treat myself like a friend and be conscious of the way I talk to myself in my head with compassion rather than judging or being super critical or worrying about others being critical. |
|  |  | When I do it it's very helpful and lovely |
|  | Sleep & relaxation | Sometimes I feel like it relaxes me so much that I come to at the end and I wonder if I took it all in or if I fell asleep? |
|  |  | I am enjoying the program although, i like some content better than others. I have been using the program before going to sleep and it has been helping me to fall asleep. I've also done it when I've woken in the middle of the night and it's helped me to fall back asleep faster. |
|  | Too busy | Very busy week, didn't have any time to do the program |
|  | Difficulties concentrating | Sometimes I find it very difficult to focus on the program and my mind wanders, it would help for more strategies for this. |
|  | Functionality | One thing I found a little tricky this week was the interface on the web browser.  It can be hard to tell where I'm up to because if I've looked at the page the completion circle is half way. If I access the audio the circle is still half full. It's only when I click to the next page it registers I've completed the mediation. There is a good chance I've accidentally clicked ahead and have missed a day, or repeated days. I'm doing the activities almost every day but there's a chance the data will be slightly out! |
| **MMM3** | Content dislikes | I found it hard to be in the moment when walking. I was a lot more distracted. |
| 8 participants in total responded |  | Find the program a bit repetitive doing the same exercise everyday |
|  |  | I enjoyed the sounds aspect to the meditation as just focusing on breathing/walking for 15 min does get boring. |
|  | Physical barrier | Had a really sore back this week where I am could hardly walk so walking meditation was very difficult |
|  | Functionality | I find it too easy to lose track of my progress. Day 30 etc doesn't mean anything to me, I would prefer to have it dated so I know when my week officially starts and which days I have missed. This would help me to stay more on track. |
|  |  | It would be great if the completed days were a different colour, ticked etc, to help visualise easily the days completed.    I have found the left foot gets quite a bit of time, presumably to get into the 'noticing', but then there's a gear change and the rest of the body is a bit raced through by comparison. |
|  | Time | Even though I haven't made time to do the program it has been on my mind to take some time for myself and check in with my mind and body |
|  |  | Found it hard this week to get time to myself and to focus |
